# Personalized Secukinumab Treatment in Patients with Plaque Psoriasis Using Model-Informed Precision Dosing

**DOI:** 10.3390/pharmaceutics16121576

**Published:** 2024-12-10

**Authors:** Karine Rodriguez-Fernandez, Javier Zarzoso-Foj, Marina Saez-Bello, Almudena Mateu-Puchades, Antonio Martorell-Calatayud, Matilde Merino-Sanjuan, Elena Gras-Colomer, Monica Climente-Marti, Victor Mangas-Sanjuan

**Affiliations:** 1 Department of Pharmacy and Pharmaceutical Technology and Parasitology, University of Valencia, 46100 Valencia, Spain; karofer@alumni.uv.es (K.R.-F.); javier.zarzoso@uv.es (J.Z.-F.); matilde.merino@uv.es (M.M.-S.); 2 Interuniversity Research Institute for Molecular Recognition and Technological Development, Polytechnic University of Valencia–University of Valencia, 46100 Valencia, Spain; 3 Pharmacy Service, Doctor Peset University Hospital, Foundation for the Promotion of Health and Biomedical Research in the Valencian Region (FISABIO), 46017 Valencia, Spain; saez_marbel@gva.es; 4 Dermatology Service, Doctor Peset University Hospital, Foundation for the Promotion of Health and Biomedical Research in the Valencian Region (FISABIO), 46017 Valencia, Spain; mateu_alm@gva.es; 5 Dermatology Service, Hospital Manises of Valencia, 46940 Manises, Spain; martorell_antcal@gva.es; 6 Pharmacy Service, Hospital Manises of Valencia, 46940 Manises, Spain; gras_ele@gva.es

**Keywords:** psoriasis, secukinumab, pharmacokinetic/pharmacodynamic

## Abstract

**Background/Objectives:** Patient care and control of inflammatory disorders, such as psoriasis, can be improved by model-informed precision dosing (MIPD) techniques based on population pharmacokinetic/pharmacodynamic (PK/PD) models. Clinical dose selection decisions based on MIPD strategies need to take account of the uncertainty associated with the individual PK/PD model parameters, which is determined by the quantity of individual observational data collected in clinical practice. **Methods:** The aim of this study was to propose an approach for personalized dosage regimens of secukinumab (SCK) in 22 Spanish patients with plaque psoriasis, whose severity level was considered moderate to severe, taking into account the uncertainty associated with individual parameters in a population-based PK/PD model. **Results:** The link between SCK serum concentrations and Psoriasis Area and Severity Index (PASI) scores was explained using an indirect response model. A maximum inhibition (I_max_) drug effect model was applied to limit the progression of psoriatic skin lesions within the turnover PD mechanism, which explains the changes in PASI scores during treatment. A first-order remission rate constant for psoriatic lesions (k_out_ = 0.11 day^−1^) was estimated. **Conclusions:** According to the MIPD strategy, 50% of patients would require an optimized regimen and 14% would require an intensified dosage regimen in comparison to current clinical treatment. This research has shown its usefulness as a tool for choosing individualized SCK dosage regimens in patients with long-lasting plaque psoriasis to improve the probability of achieving satisfactory response levels.

## 1. Introduction

Secukinumab (SCK) is a fully human immunoglobulin G1k (IgG1k) monoclonal antibody (mAb) designated for the treatment of psoriasis [1,2], a chronic autoimmune skin disorder characterized by pro-inflammatory cytokines and keratinocyte hyperproliferation [3,4,5]. Psoriasis disease is partially explained by an elevation of proinflammatory interleukin (IL)-17A, which binds to the IL-17 receptor present in keratinocytes, triggering the signaling pathway associated with inflammatory processes [6,7]. The pharmacological mechanism of action of SCK consists in selective binding to IL-17A, which diminishes its interaction with the IL-17 receptor, and therefore, reduces the inflammatory cascade [8].

Currently, SCK (Cosentyx^®^) is approved as an injection for subcutaneous (SC) use for treating plaque psoriasis, also called psoriasis vulgaris, from moderate to severe modality in adults. As indicated in the summary of product characteristics (SmPC) [9], SCK should be administered in a dose of 300 mg in weeks 0, 1, 2, 3, and 4 (induction period), and then every 4 weeks (maintenance period). For patients with a body weight of 90 kg or more, a maintenance dose of 300 mg every 2 weeks may provide additional benefit [10]. Clinical trials evaluating the efficacy of SCK in psoriasis have reported notable reductions in Psoriasis Area and Severity Index (PASI) scores, reflecting a substantial improvement in disease severity [11,12,13,14,15,16,17].

The population pharmacokinetics (PK) properties of SCK have been characterized using pooled results from six clinical trials: one phase I, three phase II, and two phase III studies in patients with psoriasis [11,12,13,18,19,20]. A two-compartment PK model with first-order absorption for SC administration and with zero-order infusion for intravenous administration successfully characterized its longitudinal PK behavior in patients with moderate to severe psoriasis [21,22]. SCK shows a terminal half-life of 27 days and slow clearance (CL) (0.19 L/day). Low central (V_2_ = 3.61 L) and peripheral (V_3_ = 2.87 L) volumes of distribution were estimated. An allometric relationship between body weight and CL and V_2_ characterized the influence of body weight on PK disposition parameters of SCK. A disease progression model incorporating a symptomatic drug effect was recently developed for SCK [23]. In this model, the proportion of patients achieving PASI 75 and PASI 90 reported in clinical trials was used as the efficacy index. 

The initial treatment with SCK is indicated by dermatologists following the labeled dosing recommendations. This procedure may not be the most favorable or harmless for all patients [24]. When the patient is in the maintenance phase of treatment with SCK because of the variable psoriasis disease progression and the fluctuation in individual response, modifications in SmPC dosage regimens become necessary. These modifications in the routine clinical practice of a dermatology service are called intensifications and optimizations. The goal of dosage regimen optimization is to either decrease the dose while keeping the dosing interval the same or retain the dose whereas the dosing interval is expanded. In contrast, dosage regimen intensification results in keeping the dose but cutting the dosing interval or increasing the dose but keeping the dosing interval. Due to the lack of a model-informed decision-making process, this strategy raises the likelihood of adverse outcomes caused by the emergence of e subtherapeutic or supratherapeutic drug concentrations [25]. Furthermore, rigorous adherence to the sample schedule is required by the individualization techniques that are frequently employed in conventional clinical practice, like those related to therapeutic drug monitoring (TDM) and clinical response. During these techniques, the drug’s exposure in patients is compared to an objective range to make modifications, and intervention usually happens only after the medication has achieved a steady state. In cases when the exposure deviates from this range, the dosage is modified based on either clinical experience or the hypothesis of proportionality in dose exposure at a steady state [26,27,28,29]. 

For dosage selection in a patient, the ideal treatment and addressing events of insufficient response, gradual depletion of efficacy, or the advent of side effects, dermatologists must adopt an adaptable approach for treating chronic psoriasis vulgaris that contemplates dosage regimens not included in the SmPC (non-labeled) and transitions between various therapeutic alternatives of the disease. These obstacles underline how crucial it is to switch in the direction of model-informed precision dosing (MIPD) techniques based on population pharmacokinetic/pharmacodynamic (PK/PD) models. Because MIPD allows for the use of any timed sample, interventions can be carried out before the initial dosage or until a steady state is attained. The procedure of intervention determines a dosage that meets a predetermined target for PK/PD [29,30,31,32,33,34]. Therapeutic results and patient safety are improved when PK/PD models are used in dosage selection for the control of chronic plaque psoriasis, since they address the interindividual variability (IIV) in therapy responses and increase precision dosing [35,36,37]. Therefore, this study aims to suggest a strategy for individualizing dosage regimens of SCK in patients with moderate to severe long-lasting psoriasis vulgaris reflecting the uncertainty associated with individual parameters obtained from a population PK/PD model. 

## 2. Materials and Methods

### 2.1. Research Design

A prospective, observational, post-authorization clinical practice follow-up study was carried out at Dr. Peset University Hospital of Valencia on Spanish patients with chronic psoriasis vulgaris from moderate to severe modality. The study’s authors certify that the methods used in the investigation comply with the moral guidelines set out by relevant institutional and national bodies that regulate the use of human subjects in research. Additionally, they guarantee adherence to the 1975 Helsinki Declaration, which underwent amendments in 2008. The Dr. Peset University Hospital Ethics Committee approved the study (protocol code VMS-SCK-2020-01 EPA-SP). Prior to beginning any study procedures, the patients’ written informed consent was obtained. The time frame for the study was July 2020–December 2022. Patients who were receiving therapy at the time of registration and had received at least one dose of SCK (Cosentyx^®^) were eligible to participate. Personalized (optimized/intensified) dose regimens were prescribed to the study participants by the dermatologist, considering their clinical responses. Pregnant women, those under the age of 18, and people with cognitive impairments were all excluded. For every patient, therapy-related information including time, dose, and treatment line were gathered. Demographic information, such as weight, sex, height, and age, were also obtained from the hospital’s electronic medical records.

### 2.2. Blood Collection and Sample Analytical Quantification

The patients received 150 or 300 mg of SCK by SC injection (abdomen or upper thigh) every 4, 5 and 6 weeks (q4w, q5w and q6w). Blood samples for PK analysis were collected using plain red vacutainer tubes immediately before SCK administration and approximately 2, 7, 14, 22, 30, and 40 days afterwards. The collected blood samples were centrifuged for 10 to 15 min at 3500–4000 rpm, and subsequently, the remaining was transferred to another tube and frozen until processing. Concentrations of SCK in serum were measured by A. Menarini Diagnostics (08918 Badalona, Barcelona, Spain) using an ISO 15189 [38] validated enzyme-linked immunosorbent assay (ELISA) in a freedom Evolyzer Tecan. The concentration range of the calibration curve was 0.2 µg/mL to 225 µg/mL.

### 2.3. Assessment of Psoriasis Area and Severity Index Score 

Patients which began SCK therapy at Dr. Peset University Hospital’s dermatological service received PASI evaluations between weeks 5 and 6 and weeks 16 and 24 after the induction period. Following this, PASI measurements were performed every 6 months. All the PASI scores that were available and registered in patients’ clinical records through the almost 1.5-year follow-up period were used for this investigation. Medical records of the patients were additionally examined to extract the baseline PASI values registered at the beginning of the SCK therapy.

### 2.4. Modeling Data Analysis

**Figure 1:**
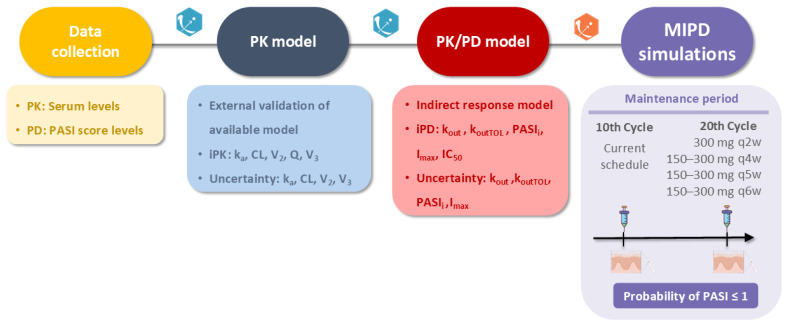
Workflow of the modeling process. PASI: Psoriasis Area and Severity Index; PD: pharmacodynamic; PK: pharmacokinetic; iPK: individual pharmacokinetic parameters; k_a_: absorption rate constant; CL: clearance; Q: intercompartmental transfer clearance; V_2_: central volume of distribution; V_3_: peripheral volume of distribution; iPD: individual pharmacodynamic parameters; k_out_: first-order reduction constant rate of psoriatic skin lesion; k_outTOLt_: first-order remission constant rate of tolerance; ${PASI}_{i}$: estimated baseline levels of PASI response; I_max_: maximum inhibition drug effect model; IC_50_: concentration of the drug needed to inhibit 50% of the response; MIPD: model-informed precision dosing; q2w: once every 2 weeks, q4w: once every 4 weeks, q5w: once every 5 weeks, q6w: once every 6 weeks.

**Figure 2:**
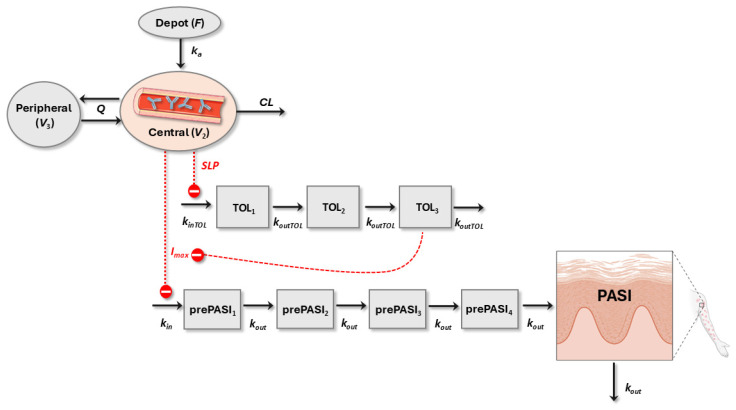
Final PK/PD model representation. *F*: bioavailability; k_in_: zero-order evolution constant rate of psoriatic skin lesion; k_inTOL_: zero-order progression constant rate of tolerance; SLP: linear drug effect model.

A summary of the modeling approach used is shown in Figure 1. The population PK parameters of the published reference model for SCK [21] were employed for simulation to determine if the available model was able to accurately represent our data. A two-compartment model with an absorption compartment through linear processes (Figure 2) represents the structural PK model. Then, a population PK/PD model was developed to characterize the time course of PASI response [39,40]. Several structural PK/PD models were proposed to characterize the time delay between PK and PD observations. Linear, maximum inhibition (I_max_), and functions for sigmoid drug effects were assessed [41]. The first section of the Supplementary Materials provides the ordinary differential equations of the PK/PD model.

The B2 method [42] was applied to estimate baseline levels of PASI response, where ${PASI}_{i}$ characterizes the individual predicted baseline level of PASI, the individual observed baseline are represented by ${PASI}_{i,0}$, and the distinguishing features between ${PASI}_{i}$ and ${PASI}_{i,0}$ are incorporated in the estimated individual random component $\eta_{i,RV}$. The mean of this random component is equal to zero, and its variance is constrained to have the same value as the residual unexplained variability (RUV) (Equation (1)). Hence, we expected that the baseline data would likewise exhibit the variability seen in the remaining data.

(1) $${PASI}_{i}={PASI}_{i,0}\cdot e^{\eta_{i,RV}}$$

A comparison of the minimum value of the objective function, a visual examination of goodness-of-fit plots, and the accuracy of model parameters as indicated by the relative standard errors (RSE) were the basis for selecting the PK/PD model. By applying simulation-based diagnostics called prediction-corrected visual predictive checks (pcVPC), the evaluation of the elected PK/PD models was completed [40,43]. The data analyses were conducted via the population approach established in the software Monolix 2024R1 (Lixoft SAS, a Simulations Plus company, Lancaster, CA, USA) [44]. The R software (version 4.4.1; http://cran.r-project.org, accessed on 15 November 2024) [45,46] was used for statistical and graphical analysis.

### 2.5. Individual Dosage Regimen Approach

The most appropriate MIPD regimen for each patient was found by simulation analysis incorporating the PK and PD individual parameters and their uncertainties, in order to investigate the performance of the final PK/PD model and its influence on clinical practice. The PK and PD individual parameter estimates and their corresponding uncertainty were obtained by computing the individual conditional distributions of PK/PD parameters in Monolix 2024R1 [47]. The degree to which PK and PD individual parameters can be accurately obtained based on the observed data and covariate value for that individual, reflecting that the individual is a member of the population for which the typical parameter value (fixed effects) and the variability (standard deviation of the random effects) were previously estimated, is known as uncertainty [47,48]. 

A Markov chain Monte Carlo (MCMC) process known as Metropolis–Hastings algorithms was used to determine individual conditional distributions ($p(\psi_{i}|y_{i})$, where $\psi_{i}$ indicates the individual parameters for individual i , and $y_{i\;}$ symbolizes the data observations for individual i. The following expression was used to sample parameter values from these distributions:

(2) $$p(\psi_{i}|y_{i})=\frac{p\left(y_{i}|\psi_{i}\right)\cdot \;p(\psi_{i})}{p(y_{i})}$$

where the constant that denotes the likelihood is $p(y_{i})$, the density function for the individual parameters is referred as $p(\psi_{i}$), and $p\left(y_{i}|\psi_{i}\right)$ reflects the conditional density function of the data when the individual parameter values are known.

In order to predict, via stochastic simulations, the PASI levels and trough concentrations at steady state (C_trough-ss_) expected at the 10th and 20th cycle (maintenance period) of treatment with SCK, one hundred clones per patient were created applying individual conditional distributions and later imputed to Simulx 2024R1 (Lixoft SAS, a Simulations Plus company) [49]. Simulations were performed as follows:

- 10th cycle: using each patient’s prescribed dosage regimen, simulations of individual dosage regimens were generated considering 10 cycles of SCK (steady-state conditions) administration
- 20th cycle: once 10 cycles were applied, 10 more cycles of SCK treatment for each patient were implemented additionally, to simulate the combination of alternative dose levels (150 and 300 mg) with different posology (q2w, q4w, q5w, and q6w) (Figure 1).

The probabilities of reaching the response aim (PASI score ≤ 1) in the 10th and 20th cycle for each patient, with each simulated dosage regimen, were calculated applying the following expression:

(3) $$Probability=\frac{n_{PASI}}{T_{PASI}}\times 100$$

The total quantity of simulated PASI score values (100 clones) corresponds to $T_{PASI}\;$, whereas $n_{PASI}$ is the number of simulated PASI score values that accomplish the response target (PASI score ≤ 1). The regimen with a probability ≥ 90% (i.e., ≥90 virtual patients) in the 20th cycle was chosen as the final dosage regimen. For methodological reasons, intensified dosing regimens were tested at the 20th cycle; a successful response was achieved even at the 10th cycle. When the response target was reached by the patient with several dosage regimens, it was selected as the final regimen, the one that was the most optimized among all. The most optimized dosage regimen stands for a lower dose while maintaining the dosing interval or equally a larger dosing interval but keeping the dose. After selecting a dosage regimen in the 20th cycle, it was compared with the clinical practice dosing regimen in each patient to determine if it was optimized or intensified, if the patients maintained their current regimen, or if it had not been possible to make a prediction for the patient. 

## 3. Results

### 3.1. Research Subjects

A total of 22 patients were included in the study. The modeling dataset consisted of 85 concentrations of SCK in samples of serum (PK), 106 individual values of PASI score (PD) and for each patient a baseline PASI (${PASI}_{i,0})$. Comorbidities, demographic and TDM data are presented in Table 1. Moreover, the current dosage regimens of the subjects who participated in the study are described as treatment characteristics in Table 1.

### 3.2. Population PK Model

The population PK model was a two-compartment model with first-order absorption and linear disposition processes that had been formerly issued by Bruin et al. [21]. The available data and patient population were then used to examine the statistical significance of the covariates enclosed in the original paper. Only body weight on CL (0.8) and V_2_ (1) were maintained through an allometric relationship. Given that most of the observations were in line with the identity line, individual characterization of the individual PK profiles was confirmed (Supplementary Figure S1).

### 3.3. Population PK/PD Model

An indirect response [50] was selected to describe the relationship between SCK concentration and PASI observations in which SCK inhibits the zero-order evolution constant rate of psoriatic skin lesion (k_in_). The inhibition of k_in_ through an I_max_ model provided a statistically significant reduction in the objective function value (*p*-value < 0.01). Other mAbs intended to treat moderate to severe chronic psoriasis vulgaris have previously used this model structure [51,52,53,54,55,56]. To capture the delay between SCK administration and observable effects, an additional chain of four turnover prePASI compartments was added, each representing a step in the disease progression (Figure 2).

The ${PASI}_{i}$ was estimated using the B2 method. Also, the value of first-order reduction constant rate of psoriatic skin lesion (k_out_) was computed. Due to difficulties during the minimization and convergence processes, the concentration of the drug needed to inhibit 50% of the response (IC_50_) could not be estimated and was fixed to a published value (9.35 mg/L) [23]. A tolerance mechanism was incorporated to account for the increase in PASI score over time during the administration of SCK in four patients (ID: 5, 10, 13, and 16). Tolerance and desensitization phenomena have been previously modeled in preclinical data for proinflammatory cytokines that determine the triggering of plaque psoriasis [57]. A turn-over mechanism including three mediator-like compartments was proposed, where the rate of progression of tolerance (k_inTOL_) is triggered by drug effects incorporated as a linear function (SLP) of the predicted levels of SCK in serum. 

**Figure 3:**
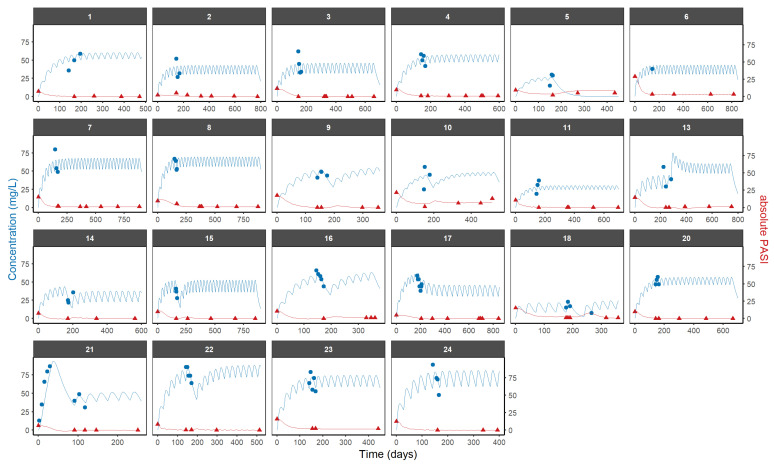
Predicted and observed PASI score (red) and SCK concentrations (blue) after the administration of SCK in patients with long-lasting psoriasis vulgaris. Individual predictions are represented by lines, and the SCK and PASI observations are represented by blue and red dots, respectively.

Individual PK and PD profiles across time are reproduced in Figure 3. According to the PK/PD model, the current framework appropriately takes into account the combined action of SCK and PASI turnover, since it can describe the longitudinal behavior of PASI at the individual level. Individual predicted vs. the observed PASI in patients with chronic plaque psoriasis and the pcVPC of the PK/PD model are shown in Supplementary Figures S2 and S3, respectively. Table 2 provides a summary of parameter estimates for the final population PK/PD model. Supplementary Tables S1 and S2 include an overview of the mean and standard deviation of the PK/PD parameters in each patient, respectively.

### 3.4. Individual Dosage Regimen Evaluation

**Figure 4:**
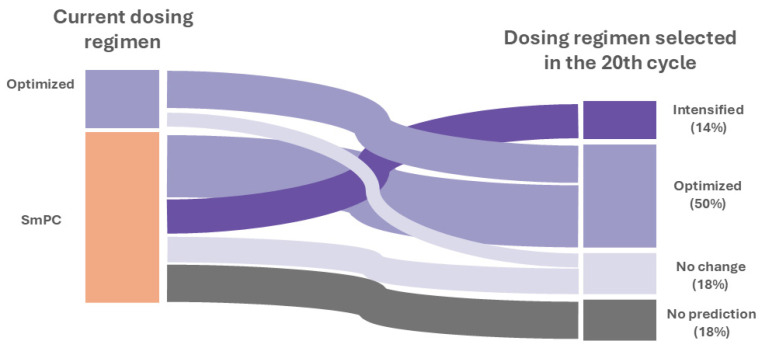
Sankey diagram of the shifts in individual dosage regimens. SmPC: summary of product characteristics.

Supplementary Figure S4 illustrates the probabilities across treatment cycles for each patient. The MIPD strategy indicated that 50% (11/22) of patients would require an optimized dose regimen, while 14% (3/22) would need an intensified regimen compared to the clinical practice regimen used during the maintenance period of treatment with SCK. The optimized patients are suggested to follow non-standard dosage regimens: 150 mg q4w for 9% (1/11), 150 mg q5w for 9% (1/11), 150 mg q6w for 18% (2/11), 300 mg q5w for 45% (5/11), and 300 mg q6w for 18% (2/11) of patients. In patients requiring intensified regimens, switching to 300 mg q2w is advised for all. For 18% (4/22) of patients, no changes to their current regimen were predicted. Furthermore, this MIPD strategy helped identify 18% (4/22) of patients who would not reach the efficacy target (90% probability of PASI ≤ 1), enabling early recognition of those at risk of therapeutic failure. Figure 4 specifies the changes from the dose regimen in clinical practice to the predicted regimen during the maintenance phase of therapy with SCK (cycle 20).

**Figure 5:**
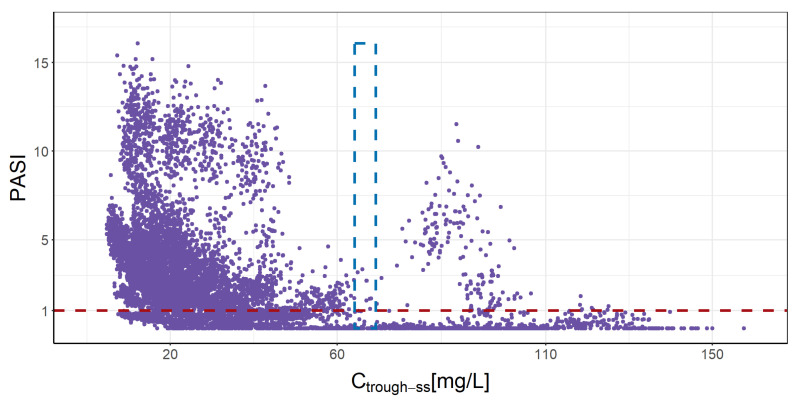
Simulated trough level and absolute PASI score for all subjects after receiving 150 mg q4w, q5w, and q6w; and 300 mg q2w, q4w, q5w and q6w of SCK SC administration in the 20th cycle. The purple dots represent the simulated PASI score and their corresponding C_trough-ss_. The blue dashed box represents the C_trough-ss_ (64.2–69.3 mg/L) that allows a PASI score ≤ 1 in 90% of patients. The red dashed line represents the PASI value of 1. C_trough-ss_: trough concentration at steady state.

Supplementary Figure S5 depicts the PK and PD simulations for each patient, comparing the current clinical practice regimen with the optimized dosing regimen determined by the 20th cycle. Figure 5 highlights the connection between absolute PASI scores and steady-state C_trough-ss_ under all tested regimens. The general findings show a non-linear association, establishing that a C_trough-ss_ varying from 64.2 to 69.3 mg/L allows a PASI score ≤ 1 in 90% of patients.

## 4. Discussion

In this study, an exposure–response model was developed for PASI, the most employed endpoint for efficacy in clinical practice patients with persistent psoriasis vulgaris. An indirect-response model was evaluated to characterize the effects of SCK, incorporating an additional series of turnover compartments for prePASI and a tolerance mechanism. This approach was based on the expectation of a time lag between the peak concentrations of SCK and the maximum drug effect on psoriatic lesions, represented by PASI scores. Since SCK inhibits the IL-17A, the exposure–efficacy model represented SCK as having an inhibitory pharmacological effect on the development of psoriatic lesions following drug administration. However, we are aware of the drawbacks in the developed PK/PD model reflected in the low precision in parameter estimation of the tolerance mechanism (RSE of k_outTOL_ and RSE of k_outTOL_ IIV), which is only activated in four patients (5, 10, 13, and 16).

The cornerstone of this study was the development of a procedure for tailoring dosing approaches of SCK in Spanish patients with moderate to severe long-lasting psoriasis vulgaris, based on the uncertainty surrounding individual parameter estimates from a population PK/PD model. Moreover, by integrating uncertainty into the simulation stage designed for personalized dosing regimen choice, it becomes possible to assess the confidence level of model-informed forecasts in clinical practice, offering an approach with probabilistic character for MIPD of SCK. Among the patients needing a modification to their dosage regimen, an optimized dosage regimen is suggested in 50% of them. In almost all optimized patients (91%), the optimization should be to a non-labeled dosage regimen, with 300 mg q5w being recommended in 45% of patients. This adjustment would lead to a decrease of 3 doses for each year, meaning that as an alternative to receiving 14 doses in the following years after the first year of treatment with SKC according to the SmPC dosing guidelines, patients would receive 11 doses. Corresponding to the health information database for medicines and pharmacy products [58], the cost of Cosentyx^®^ 300 mg injection in a pre-filled syringe is EUR 1246.98. With the optimization of SCK therapy, the annual cost per patient would amount to EUR 13717, resulting in savings of 21%. Additionally, fewer medication purchases and a smaller quantity of administrations would decrease the threat of injection site reactions. 

In contrast to the clinical practice’s dose regimen schedule, intensification was recommended in 14% of the subjects. Because in these patients the I_max_ values were lower (1.06–1.13) than the typical value (1.19), a higher level of SCK dose would be required to achieve a successful therapeutic response. Note that most of the included patients required a dose regimen optimization following the implementation of our MIPD method in SCK. Consequently, since patients are more likely to be within the appropriate treatment response range, it is possible to enhance patient welfare and health system administration. In 18% of subjects, the efficacy endpoint (90% probability of PASI ≤ 1) was not obtained. Therefore, the MIPD strategy proposed in this investigation implies a benefit for identifying patients who are not responding to treatment in the beginning. In this way, the management of plaque psoriasis can be enhanced in these individuals, and unnecessary costs associated with treating them in subsequent cycles could be avoided. Furthermore, their k_out_ values are lower (0.07) than the typically observed (0.11), which might be used as a cutoff point for distinguishing patients who are unlikely to respond optimally to SCK. 

The full data of SCK concentrations and PASI scores that were acquirable in each patient included in this study were applied to estimate the individual PK/PD parameters. An adaptable process might be employed to apply the suggested MIPD approach into practice. To make sure the model continues to be accurate and still represent the patient’s progressing state, this approach would involve a refining process of the individual parameters as novel data points appear. In this study, the importance of collecting samples of absolute PASI from patients throughout the initial weeks of treatment to accurately determine their individual PD parameters is highlighted. The significance of both PK and PD parameters in ensuring effective treatment outcomes is demonstrated by the non-linear relationship between the simulated absolute PASI and C_trough-ss_ of SCK (Figure 5). While it is feasible to set general exposure values corresponding to an absolute PASI, the use of this MIPD approach to personalize treatment can lead to a higher proportion of patients reaching efficient dosage regimens, thereby minimizing treatment failure among non-responders. 

A more exigent C_trough-ss_ range (64.2–69.3 mg/L) was proposed in this study to accomplish in 90% of patients an absolute PASI ≤ 1. A previously reported C_trough-ss_ range (12.9–62.9 mg/L) was established in a prior population PK modeling investigation [21], which included individuals on a 300 mg q4w dosage regimen, and it was produced using simulated data from clinical trials in patients who were only beginning therapy with SCK, in which the efficacy objective was to achieve in week 12 the PASI 75. Conversely, the C_trough-ss_ range found in this research stand for a more rigorous PD endpoint (PASI ≤ 1 or PASI 99) from patients in routine clinical practice. Also, the proposed C_trough-ss_ range aligns with optimal response criteria consistent with current clinical guidelines (PASI ≤ 1), intended to enhance the quality of life and clinical results for those with persistent psoriasis vulgaris [59,60].

This research is based on PK/PD models previously reported. Nevertheless, our work is an example of how to effectively use established knowledge. However, we have gone one step further in the description of the relationship among SCK concentrations and patient responses measured by absolute PASI under real-world circumstances. In addition, this investigation exemplifies individualized precision dosing, representing a more thorough and effective application of precision dosing approaches by identifying, characterizing, and quantifying the different causes of variability in drug response via PK/PD modeling [25,35]. While gaining a deeper comprehension of PK/PD interrelation in particular patient populations during clinical studies can aid in identifying precise dosing objectives, patients from clinical practice are often much more distinct than those in supervised study environments. As a result, efficient post-marketing monitoring is crucial for investigating distinctive precision-dosing aims [61]. Improvements in methods for gathering and analyzing data from real-world patients present chances to discover novel precision-dosing procedures and improve those confirmed in clinical trials. These techniques facilitate the ongoing renewing and enhancement of dosing strategies to more effectively address patient requirements [35,62]. That is why our approach, which is based on MIPD under real-world circumstances, may be a first effort to guarantee the most accurate individual dosage for patients with psoriasis vulgaris receiving SCK. 

Some limitations have been played against our investigation. Firstly, as a consequence of the study design conditions, there was a small patient sample size. Secondly, many PK/PD observations were either missing or were captured very distant in time (sparse data), resulting in a small number of points per patient. Because of that, the progression of psoriasis vulgaris or unmeasured physiological transformations may have straightforwardly affected the outcomes obtained. Looking ahead, similar models could be incorporated into a provisional distribution dashboard system for enhanced analysis [63,64]. Regarding the proposed optimization with the unlabeled dosage regimen of 300 mg q5w, and despite its advantages in terms of efficiency and safety, sometimes such optimizations may not be carried out because it would mean extending the frequency of administration by one more week. Changing the dosage could cause some confusion in, for example, elderly patients, patients who are seriously ill at the start of SCK therapy, and patients who have been heavily pretreated with multi-resistant forms. Furthermore, we incorporated tolerance mechanisms because we first developed a PKPD model with all the experimental evidence and then conducted an MIPD approach for evaluating the optimal dosing regimen. Therefore, the tolerance mechanisms could not be anticipated in a traditional TDM approach for prospective prediction without measured PK and PD levels.

The results of this study confirm the recommendations already formulated by other authors, who propose incorporating alternatives for different dosing schemes based on MIPD and/or TDM into the SmPC [65]. This proposal could enhance treatment management in chronic diseases, such as plaque psoriasis, and could facilitate real-time predictions of treatment responses, helping healthcare providers make informed decisions regarding dosing modifications and therapy transitions for SCK.

## 5. Conclusions

In conclusion, this study represents a significant initial step for biologists to implement MIPD that targets the IL routes in long-lasting psoriasis vulgaris, an area that has been largely understudied. We propose a methodology to personalize dosing strategies for SCK, which considers the uncertainty of individual parameters within a population PK/PD model to enhance the likelihood of succeeding targeted clinical effects in patients with psoriasis vulgaris from moderate to severe modality. Future research should focus on applying our approach to a larger group of real-world patients and validating the proposed dosing strategies. 

## Figures and Tables

**Table 1:** Overview of the PK/PD experimental data and patient characteristics gathered within TDM.

			Mean ± SD		Range	n (%)
Demographic data						
Body weight (kg)			74.5 ± 15		46–97	
Height (m)			1.7 ± 0.09		1.54–1.85	
BMI (kg/m^2^)			25.5 ± 4.9		16–41	
Age (years)			50.07 ± 13.4		28–76	
Gender (male)						14 (64)
Treatment period (years)			2 ± 1.7		0.005–5.1	
Biological “naive”						19 (86)
Comorbidities						
AHT						5 (23)
Dyslipidemia						7 (32)
Diabetes						3 (14)
Obesity						2 (9.1)
Psoriatic arthropathy						5 (23)
Non-alcoholic fatty liver						1 (4.5)
Anxious–depressive disorder						2 (9.1)
Others						11 (50)
TDM data						
Total of patients						22
SCK concentration (mg/L)			48.2 ± 18.5		7.4–89	85
PASI (no units)			1 ± 1.7		0–12.3	106
${PASI}_{i,0}$			11.6 ± 5.8		2–27.5	22
Clinical practice treatment characteristics						
SmPC		Optimized			Summary	
300 mg q4w	18	150 mg q4w		2	SmPC	18 (82%)
		300 mg q5w		1	Optimized	4 (18%)
		300 mg q6w		1	Intensified	none

SD: standard deviation; BMI: body mass index; AHT: arterial hypertension; TDM: therapeutic drug monitoring; SCK: secukinumab; PASI: Psoriasis Area and Severity Index; SmPC: summary of product characteristics; q4w: once every 4 weeks; q5w: once every 5 weeks; q6w: once every 6 weeks.

**Table 2:** Population PK/PD estimates in patients with chronic plaque psoriasis after administration of SCK.

Parameter (Units)	Value	RSE (%)
Fixed-effect		
k_out_ (day^−1^)	0.11	26.1
k_outTOL_ (day^−1^)	0.003	106
I_max_	1.19	1.88
IC_50_ (mg/L)	9.35 FIX	
Inter-individual variability		
k_out_ (%)	91.3	20
k_outTOL_ (%)	37.15	431
I_max_ (%)	7.63	18
Residual unexplained variability		
Error (%)	0.76	9.2

RSE: relative standard error; k_out_: first-order reduction constant rate of psoriatic skin lesion; I_max_: maximum inhibition drug effect; k_outTOLt_: first-order remission constant rate of tolerance; IC_50_: concentration of the drug needed to inhibit 50% of the response.

## Data Availability

The data presented in this study are available on request from the corresponding author due to ethical and legal restrictions.

## Supplementary Materials

The following supporting information can be downloaded at: https://www.mdpi.com/article/10.3390/pharmaceutics16121576/s1, Ordinary differential equations for the PK/PD model of SCK and absolute PASI; Figure S1: Individually predicted vs. the observed concentrations of SCK in patients with chronic psoriasis vulgaris; Figure S2: Individually predicted vs. the observed PASI in patients with chronic psoriasis vulgaris; Figure S3: Prediction-corrected visual predictive check obtained from one thousand simulated studies using the selected population PK/PD model; Table S1: Means of the individual PK/PD parameters drawn from the conditional distribution task in Monolix; Table S2: Standard deviation of the individual PK/PD parameters drawn from the conditional distribution task in Monolix; Figure S4: Bar plot of 100 simulated absolute PASI for each patient after SCK administration at cycles 10 and 20, using the individual parameters from the final population PK/PD model and their uncertainties; Figure S5: PK and PD simulations with the current dosage regimen from clinical practice and the individual optimal dosing regimen established after simulations in 20th cycle for each patient.

## Abbreviations

AHT	Arterial hypertension
BMI	Body mass index
C_trough-ss_	Trough concentration at steady state
CL	Clearance
ELISA	Enzyme-linked immunosorbent assay
F	Bioavailability
IC_50_	Concentration of the drug needed to inhibit 50% of the response
I_max_	Maximum inhibition
IIV	Interindividual variability
IL	Interleukin
k_a_	Absorption rate constant
k_in_	Zero-order evolution constant rate of psoriatic skin lesion
k_inTOL_	Zero-order progression constant rate of tolerance
k_out_	First-order reduction constant rate of psoriatic skin lesion
k_outTOL_	First-order remission constant rate of tolerance
mAb	Monoclonal antibody
MCMC	Markov chain Monte Carlo
MIPD	Model-informed precision dosing
PASI	Psoriasis Area and Severity Index
${PASI}_{i}$	Estimated baseline levels of PASI response
${PASI}_{i,0}$	Individual observed baseline levels of PASI response
pcVPC	Prediction-corrected visual predictive checks
PD	Pharmacodynamic
PK	Pharmacokinetic
PK/PD	Pharmacokinetic/pharmacodynamic
Q	Intercompartmental transfer clearance
q2w	Once every 2 weeks
q4w	Once every 4 weeks
q5w	Once every 5 weeks
q6w	Once every 6 weeks
RSE	Relative standard error
RUV	Residual unexplained variability
SC	Subcutaneous
SD	Standard deviation
SmPC	Summary of product characteristics
SLP	Linear drug effect model
TDM	Therapeutic drug monitoring
SCK	Secukinumab
V_2_	Central volume of distribution
V_3_	Peripheral volume of distribution
