# Supplementary material for: Personalized Secukinumab Treatment in Patients with Plaque Psoriasis Using Model-Informed Precision Dosing
Source: Pharmaceutics. 2024 Dec 10;16(12):1576. doi: 10.3390/pharmaceutics16121576 (PMC11678170; doi:10.3390/pharmaceutics16121576)
Supplement: Supplementary file 1 [file pharmaceutics-16-01576-s001.zip › pharmaceutics-3317641-supplementary.pdf]

Supplementary materials

Personalized Secukinumab Treatment in Patients with Plaque Psoriasis Using Model-Informed Precision Dosing

Karine Rodriguez-Fernandez [1,2,†], Javier Zarzoso-Foj [1,2,†], Marina Saez-Bello [3], Almudena Mateu-Puchades [4], Antonio Martorell-Calatayud [5], Matilde Merino-Sanjuan [1,2], Elena Gras-Colomer [6], Monica Climente-Marti [3,*] and Victor Mangas-Sanjuan [1,2,*]

[1] Department of Pharmacy and Pharmaceutical Technology and Parasitology, University of Valencia, 46100 Valencia, Spain
[2] Interuniversity Research Institute for Molecular Recognition and Technological Development, Polytechnic University of Valencia–University of Valencia, 46100 Valencia, Spain
[3] Pharmacy Service, Doctor Peset University Hospital, Foundation for the Promotion of Health and Biomedical Research in the Valencian Region (FISABIO), 46017 Valencia, Spain
[4] Dermatology Service, Doctor Peset University Hospital, Foundation for the Promotion of Health and Biomedical Research in the Valencian Region (FISABIO), 46017 Valencia, Spain
[5] Dermatology Service, Hospital Manises of Valencia, 46940 Manises, Spain
[6] Pharmacy Service, Hospital Manises of Valencia, 46940 Manises, Spain
* Correspondence: climente_mon@gva.es (M.C.-M.); victor.mangas@uv.es (V.M.-S.); Tel.: +34-963543351
† These authors contributed equally to this work.

Ordinary differential equations for the PK/PD model of SCK and absolute PASI for patients without tolerance mechanism.

$$\frac{dA}{dt} = -k_a \cdot A$$

$$\frac{dC_C}{dt} = k_a \cdot A - CL \cdot C_C + Q \cdot (C_p - C_c)$$

$$\frac{dC_P}{dt} = -Q \cdot (C_p - C_c)$$

$$\frac{dprePASI_1}{dt} = k_{in} \cdot \left(1 - \frac{I_{max} \cdot C_C}{(IC_{50} + C_C)}\right) - k_{out} \cdot prePASI_1$$

$$\frac{dprePASI_2}{dt} = k_{out} \cdot prePASI_1 - k_{out} \cdot prePASI_2$$

$$\frac{dprePASI_3}{dt} = k_{out} \cdot prePASI_2 - k_{out} \cdot prePASI_3$$

$$\frac{dprePASI_4}{dt} = k_{out} \cdot prePASI_3 - k_{out} \cdot prePASI_4$$

$$\frac{dPASI}{dt} = k_{out} \cdot prePASI_4 - k_{out} \cdot PASI$$

where k_a: absorption rate constant; A: amount of SCK in the depot compartment.; CL: clearance; C_c: concentration of SCK in the central compartment; Q: intercompartmental transfer clearance; C_p: concentration of SCK in the peripheral compartment; $prePASI_{1-4}$: pre Psoriasis Area and Severity Index compartment; k_{in}: zero-order progression constant rate of psoriatic skin lesion; I_{max}: maximum inhibition drug effect model; IC_{50}: concentration of SCK needed to inhibit 50% of the response; k_{out}: first-order remission constant rate of psoriatic skin lesion; PASI: Psoriasis Area and Severity Index compartment.

Ordinary differential equations for the PK/PD model of SCK and PASI for patients with tolerance mechanisms

$$\frac{dA}{dt} = -k_a \cdot A$$

$$\frac{dC_C}{dt} = k_a \cdot A - CL \cdot C_C + Q \cdot (C_p - C_c)$$

$$\frac{dC_P}{dt} = -Q \cdot (C_p - C_c)$$

$$\frac{dTOL_1}{dt} = k_{in_{TOL}} \cdot (1 - SLP \cdot C_C) - k_{out_{TOL}} \cdot TOL_1$$

$$\frac{dTOL_2}{dt} = k_{out_{TOL}} \cdot TOL_1 - k_{out_{TOL}} \cdot TOL_2$$

$$\frac{dTOL_3}{dt} = k_{out_{TOL}} \cdot TOL_2 - k_{out_{TOL}} \cdot TOL_3$$

$$\frac{dprePASI_1}{dt} = k_{in} \cdot \left(1 - \frac{(I_{max} \div TOL_3) \cdot C_C}{(IC_{50} + C_C)}\right) - k_{out} \cdot prePASI_1$$

$$\frac{dprePASI_2}{dt} = k_{out} \cdot prePASI_1 - k_{out} \cdot prePASI_2$$

$$\frac{dprePASI_3}{dt} = k_{out} \cdot prePASI_2 - k_{out} \cdot prePASI_3$$

$$\frac{dprePASI_4}{dt} = k_{out} \cdot prePASI_3 - k_{out} \cdot prePASI_4$$

$$\frac{dPASI}{dt} = k_{out} \cdot prePASI_4 - k_{out} \cdot PASI$$

where k_a: absorption rate constant; A: amount of SCK in the depot compartment.; CL: clearance; C_c: concentration of SCK in the central compartment; Q: intercompartmental transfer clearance; C_P: concentration of SCK in the peripheral compartment; TOL_{1-3}: tolerance compartment; k_{inTOL}: zero-order progression constant rate of tolerance; $k_{outTOLt}$: first-order remission constant rate of tolerance; $prePASI_{1-4}$: pre Psoriasis Area and Severity Index compartment; k_{in}: zero-order progression constant rate of psoriatic skin lesion; I_{max}: maximum inhibition drug effect model; IC_{50}: concentration of SCK needed to inhibit 50% of the response; k_{out}: first-order remission constant rate of psoriatic skin lesion; PASI: Psoriasis Area and Severity Index compartment.

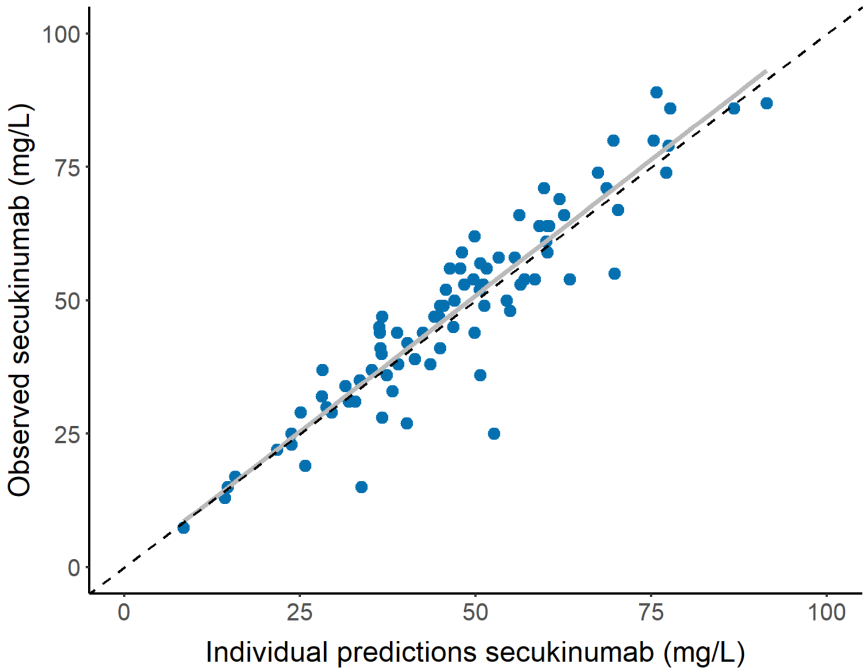

Figure S1. Individual predicted vs the observed concentrations of SCK in patients with chronic psoriasis vulgaris.

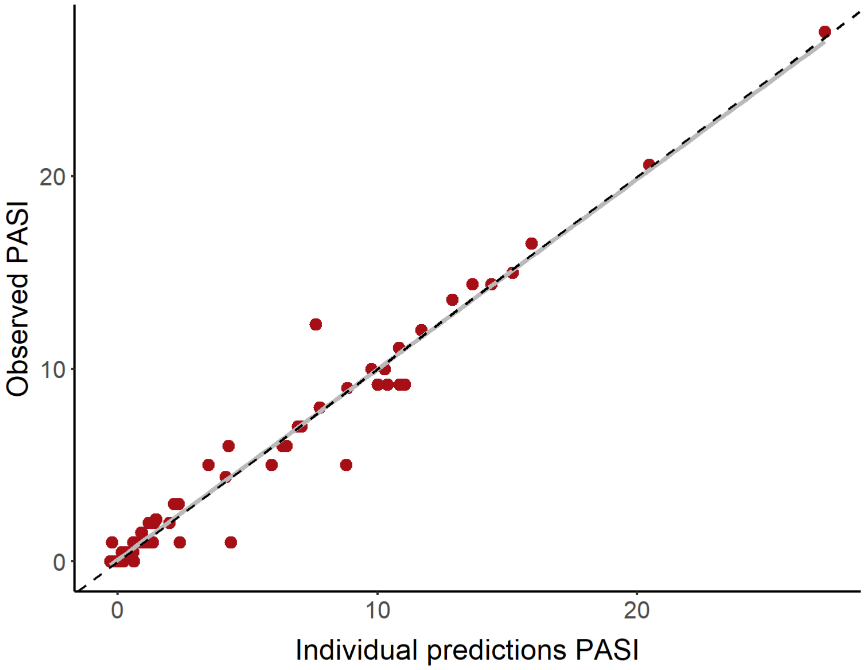

Figure S2. Individual predicted vs the observed PASI in patients with chronic psoriasis vulgaris.

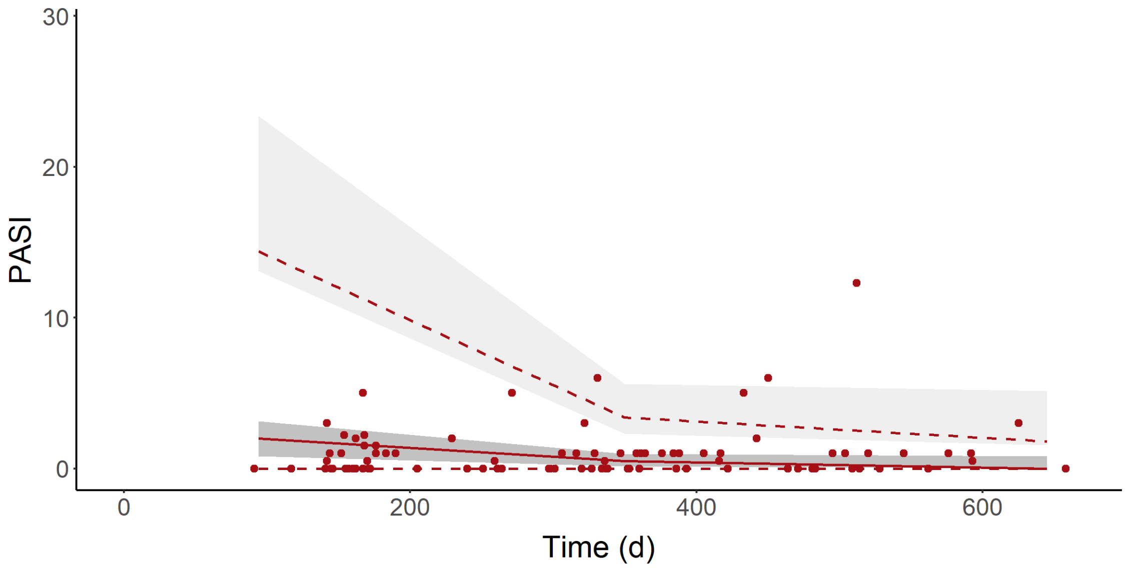

Figure S3. Prediction-corrected visual predictive check obtained from one thousand simulated studies using the selected population PK/PD model. Points represent the observed serum SCK concentrations; lines, 2.5th, 50th, and 95th percentiles of the simulated data. Shaded areas represent the 95% prediction intervals of the 5th, 50th, and 95th percentiles of the simulated studies.

Table S1. Mean of the individual PK/PD parameters drawn from the conditional distribution task in Monolix. For all patients F = 72.9 % and IC_{50} = 9.35 mg/L.

Subjects	PK					PD			
	k_a (day^{-1})	CL (L/d)	V_2 (L)	Q (L/d)	V_3 (L)	k_{out} (day^{-1})	k_{outTOL} (day^{-1})	I_{max}	$PASI_i$
1	0.20	0.15	3.72	0.40	2.93	0.47	0.0027	1.17	7.05
2	0.18	0.20	4.34	0.45	3.46	0.02	0.0033	1.10	1.98
3	0.20	0.11	1.99	0.26	1.71	0.09	0.0033	1.27	10.66
4	0.18	0.17	4.16	0.44	3.35	0.13	0.0033	1.13	10.78
5	0.18	0.26	4.60	0.47	3.62	0.07	0.0033	1.17	8.81
6	0.18	0.18	3.37	0.37	2.68	0.12	0.0033	1.13	27.40
7	0.19	0.15	3.31	0.37	2.68	0.08	0.0033	1.12	14.37
8	0.18	0.14	2.80	0.32	2.24	0.04	0.0033	1.09	10.99
9	0.18	0.17	4.34	0.45	3.37	0.25	0.0033	1.21	15.88
10	0.17	0.17	4.38	0.45	3.39	0.14	0.0044	1.20	20.38
11	0.19	0.17	3.22	0.38	2.80	0.12	0.0035	1.42	9.73
13	0.19	0.17	3.67	0.39	2.94	0.07	0.0028	1.34	12.76
14	0.20	0.19	3.52	0.39	2.82	0.09	0.0031	1.29	6.92
15	0.19	0.17	2.69	0.32	2.15	0.11	0.0032	1.20	10.35
16	0.17	0.15	4.19	0.44	3.42	0.12	0.0035	1.22	10.22
17	0.16	0.15	3.00	0.31	2.19	0.06	0.0030	1.27	4.12
18	0.18	0.26	4.00	0.42	3.17	0.15	0.0033	1.27	13.81
20	0.17	0.20	3.92	0.43	3.26	0.29	0.0033	1.24	9.95
21	0.34	0.16	3.38	0.42	3.17	0.18	0.0033	1.21	6.27
22	0.17	0.11	2.79	0.32	2.20	0.07	0.0034	1.13	7.73
23	0.18	0.13	2.63	0.30	2.09	0.09	0.0033	1.06	15.13
24	0.16	0.12	2.67	0.31	2.11	0.20	0.0033	1.15	11.59

k_a: absorption rate constant; F: bioavailability CL: clearance; Q: intercompartmental transfer clearance; V_2: central volume of distribution; V_3: peripheral volume of distribution; k_{out}: first-order remission constant rate of psoriatic skin lesion; $k_{outTOLt}$: first-order remission constant rate of tolerance; I_{max}: maximum inhibition drug effect model; $PASI_i$: estimated baseline levels of PASI response.

Table S2. Standard deviation of the individual PK/PD parameters drawn from the conditional distribution task in Monolix.

Subjects	PK					PD			
	k_a (day^{-1})	CL (L/d)	V_2 (L)	Q (L/d)	V_3 (L)	k_{out} (day^{-1})	k_{outTOL} (day^{-1})	I_{max}	$PASI_i$
1	0.023	0.006	0.284	0	0.073	0.216	0.0012	0.011	0.097
2	0.024	0.013	0.370	0	0.100	0.006	0.0012	0.098	0.106
3	0.018	0.014	0.287	0	0.088	0.034	0.0012	0.034	0.167
4	0.023	0.010	0.374	0	0.102	0.152	0.0013	0.023	0.103
5	0.021	0.020	0.405	0	0.105	0.131	0.0014	0.062	0.089
6	0.021	0.015	0.301	0	0.078	0.178	0.0012	0.028	0.100
7	0.025	0.009	0.283	0	0.077	0.131	0.0013	0.019	0.101
8	0.026	0.008	0.255	0	0.070	0.011	0.0013	0.022	0.100
9	0.022	0.004	0.664	0	0.119	0.063	0.0011	0.005	0.068
10	0.019	0.010	0.433	0	0.112	0.163	0.0012	0.059	0.098
11	0.028	0.010	0.207	0	0.091	0.016	0.0012	0.026	0.074
13	0.018	0.007	0.269	0	0.084	0.054	0.0012	0.035	0.119
14	0.022	0.008	0.279	0	0.056	0.075	0.0013	0.021	0.081
15	0.018	0.012	0.200	0	0.064	0.012	0.0014	0.014	0.065
16	0.031	0.006	0.372	0	0.175	0.090	0.0006	0.027	0.100
17	0.023	0.007	0.207	0	0.077	0.035	0.0013	0.015	0.107
18	0.021	0.021	0.368	0	0.089	0.087	0.0013	0.048	0.095
20	0.012	0.022	0.206	0	0.096	0.019	0.0013	0.027	0.092
21	0.022	0.010	0.179	0	0.097	0.053	0.0013	0.017	0.145
22	0.022	0.005	0.254	0	0.074	0.054	0.0012	0.024	0.110
23	0.021	0.007	0.245	0	0.065	0.103	0.0013	0.030	0.101
24	0.006	0.005	0.109	0	0.047	0.169	0.0012	0.006	0.123

k_a: absorption rate constant; F: bioavailability CL: clearance; Q: intercompartmental transfer clearance; V_2: central volume of distribution; V_3: peripheral volume of distribution; k_{out}: first-order remission constant rate of psoriatic skin lesion; $k_{outTOLt}$: first-order remission constant rate of tolerance; I_{max}: maximum inhibition drug effect model; $PASI_i$: estimated baseline levels of PASI response.

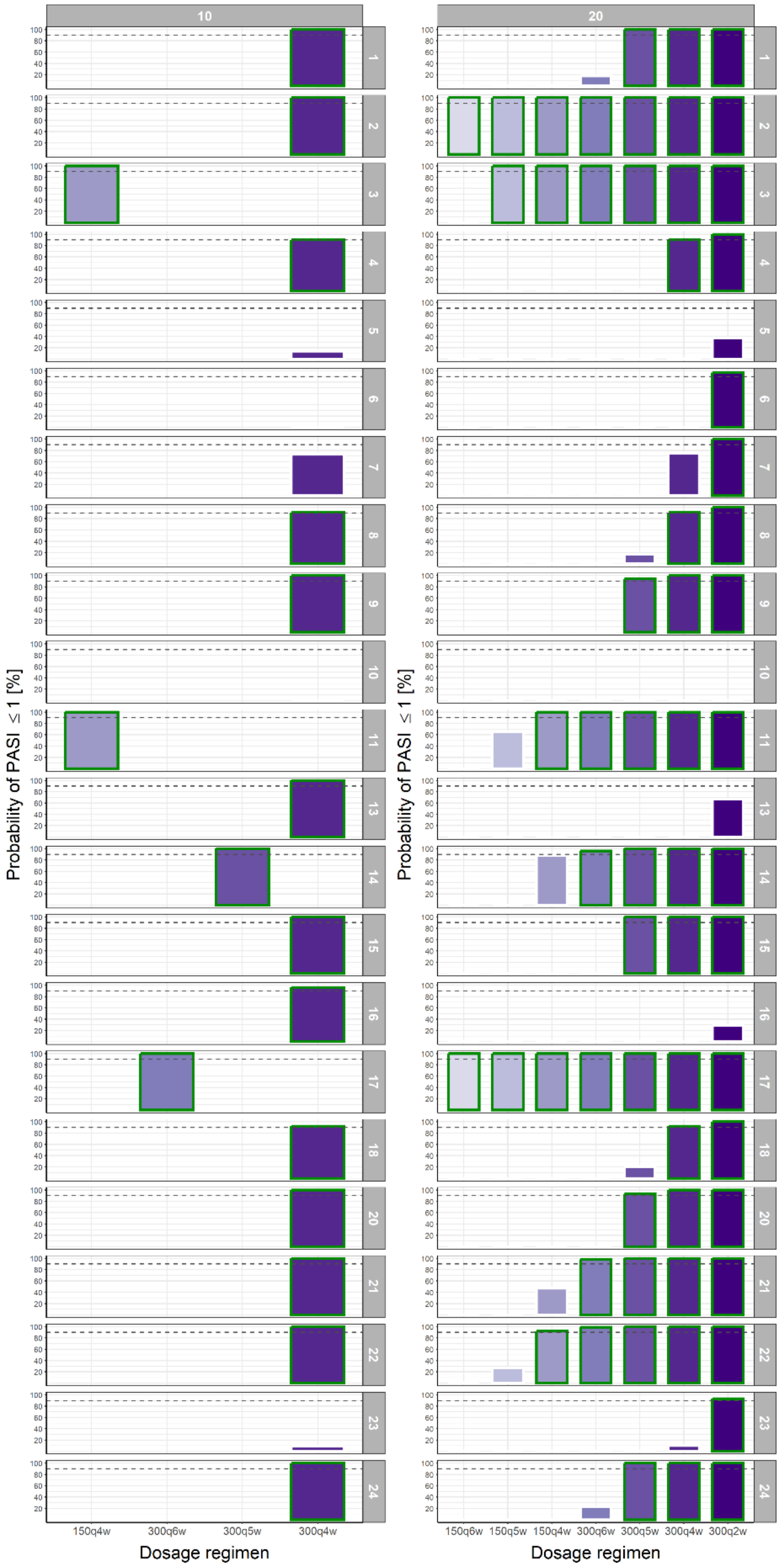

Figure S4. Bar plot of 100 simulated absolute PASI for each patient after SCK administration at cycles 10 and 20, using the individual parameters from the final population PK/PD model and their uncertainties. In green it is represented the probability ≥ 90% of PASI level ≤1. The dose regimens in which a probability ≥ 90% is reached are indicated in green.

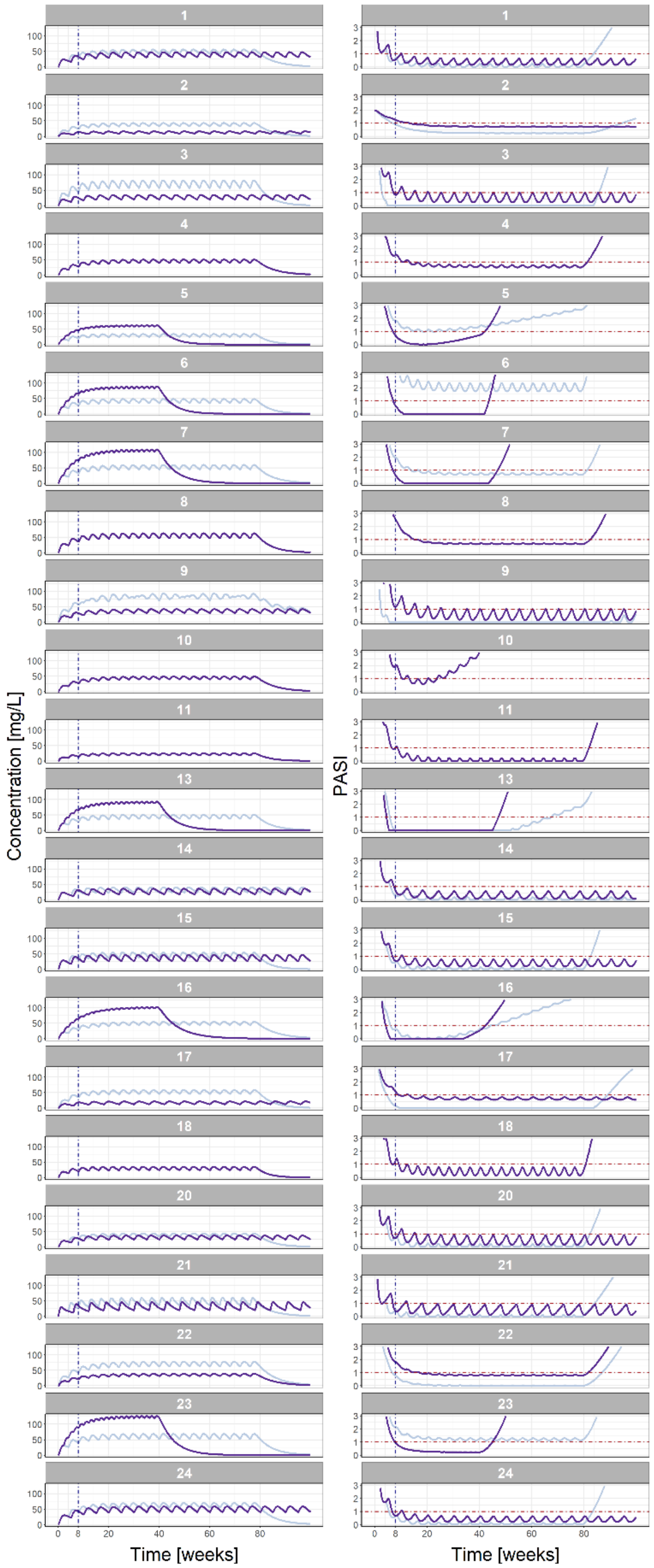

Figure S5. PK and PD simulations with the current dosage regimen from clinical practice (blue) and the individual optimal dosing regimen established after simulations in the 20th cycle (purple) for each patient to compare the plasmatic concentration tendency (left panel) and the PASI score behavior (right panel) according to the dosage regimen that the patient receives. The vertical dashed line represents the starting point of the maintenance period at week 8. The horizontal dashed line represents the PASI value of 1.
